# Accelerating open modification spectral library searching on tensor core in high-dimensional space

**DOI:** 10.1093/bioinformatics/btad404

**Published:** 2023-06-27

**Authors:** Jaeyoung Kang, Weihong Xu, Wout Bittremieux, Niema Moshiri, Tajana Rosing

**Affiliations:** Department of Electrical and Computer Engineering, University of California San Diego, San Diego, CA 92093, United States; Department of Computer Science and Engineering, University of California San Diego, San Diego, CA 92093, United States; Department of Computer Science, University of Antwerp, Antwerpen 2020, Belgium; Department of Computer Science and Engineering, University of California San Diego, San Diego, CA 92093, United States; Department of Computer Science and Engineering, University of California San Diego, San Diego, CA 92093, United States

## Abstract

**Motivation:**

Driven by technological advances, the throughput and cost of mass spectrometry (MS) proteomics experiments have improved by orders of magnitude in recent decades. Spectral library searching is a common approach to annotating experimental mass spectra by matching them against large libraries of reference spectra corresponding to known peptides. An important disadvantage, however, is that only peptides included in the spectral library can be found, whereas novel peptides, such as those with unexpected post-translational modifications (PTMs), will remain unknown. Open modification searching (OMS) is an increasingly popular approach to annotate modified peptides based on partial matches against their unmodified counterparts. Unfortunately, this leads to very large search spaces and excessive runtimes, which is especially problematic considering the continuously increasing sizes of MS proteomics datasets.

**Results:**

We propose an OMS algorithm, called HOMS-TC, that fully exploits parallelism in the entire pipeline of spectral library searching. We designed a new highly parallel encoding method based on the principle of hyperdimensional computing to encode mass spectral data to hypervectors while minimizing information loss. This process can be easily parallelized since each dimension is calculated independently. HOMS-TC processes two stages of existing cascade search in parallel and selects the most similar spectra while considering PTMs. We accelerate HOMS-TC on NVIDIA’s tensor core units, which is emerging and readily available in the recent graphics processing unit (GPU). Our evaluation shows that HOMS-TC is 31× faster on average than alternative search engines and provides comparable accuracy to competing search tools.

**Availability and implementation:**

HOMS-TC is freely available under the Apache 2.0 license as an open-source software project at https://github.com/tycheyoung/homs-tc.

## 1 Introduction

Mass spectrometry (MS) is a powerful analytical technique to identify and quantify peptides and proteins present in complex biological samples. A common strategy to analyze data from shotgun proteomics experiments is using spectral library searching, which matches experimental MS/MS spectra to reference MS/MS spectra corresponding to known peptides and transfers peptide labels to high-scoring matches (Griss 2016, Shao and Lam 2017). An important disadvantage of this approach, however, is that only peptides that are included in the spectral library can be found, while novel peptides will remain unknown. Even though spectral libraries are becoming increasingly comprehensive, they cannot provide coverage of the full proteome. Notably, current spectral libraries predominantly contain unmodified peptides, whereas a significant portion of experimental spectra that remain unannotated might correspond to peptides that include post-translational modifications (PTMs) (Chick *et al.* 2015).

An increasingly popular approach to identifying modified peptides is open modification searching (OMS). Unlike standard spectral library searching, which only compares experimental MS/MS spectra to library candidates with a similar precursor mass, OMS matches spectra irrespective of their precursor mass. This makes it possible to compare spectra corresponding to modified and unmodified peptides, even when their precursor masses differ due to PTMs. An important downside of OMS is that, because the precursor mass filter can no longer be used and instead each experimental spectrum has to be compared against the full spectral library, it suffers from a very large search space, which can lead to excessive runtimes. This is especially relevant as available spectral libraries have grown significantly in size over the past few years (Griss *et al.* 2013, Griss *et al.* 2016, Wang *et al.* 2018, Xu *et al.* 2022). For example, PRIDE-Cluster (Griss *et al.*, 2013, 2016) and MassIVE-KB (Wang *et al.* 2018) have been generated by repository-scale reprocessing of public data on the PRIDE (Martens *et al.* 2005) and MassIVE data repositories, respectively, and consist of millions of reference MS/MS spectra.

To cope with the computational demand, various tools that use algorithmic advances to efficiently process large search spaces have been proposed, such as MSFragger (Kong *et al.* 2017), Open-pFind (Chi *et al.* 2018), TagGraph (Devabhaktuni *et al.* 2019), MetaMorpheus (Solntsev *et al.* 2018), ANN-SoLo (Bittremieux *et al.* 2018, 2019), and others. Some tools can use specialized hardware, such as graphics processing units (GPUs), to speed up OMS (Bittremieux *et al.* 2019). Although spectrum identification is generally not the bottleneck compared with the data acquisition time, because there is a strong effort to reduce experimental runtimes, e.g. by using very short liquid chromatography gradients (Messner *et al.* 2021), computational efficiency is still an important point of attention. Additionally, as the amount of MS data that is available in public data repositories, such as PRIDE (Martens *et al.* 2005) and MassIVE, continuously keeps growing, efficient bioinformatics tools are essential for large-scale public data reanalysis efforts. Unfortunately, however, due to the complexities of spectral data processing, in practice, most OMS tools under-utilize the hardware resources and still suffer from long runtimes.

In this work, we propose Hyperdimensional Open Modification Search with Tensor Core (HOMS-TC) acceleration, a novel spectral library searching framework that supports OMS. Our solution redesigns the MS/MS spectral matching algorithm based on the principle of hyperdimensional computing (HDC). HDC is designed to mimic the efficiency of human memory in pattern-oriented computations by representing data with high-dimensional (HD) vectors, called *hypervectors* (HVs) (Kanerva 1988, 2009). HDC has been used to enable parallel processing of pattern-matching tasks, such as sequence alignment (Kim *et al.* 2020, Zou *et al.* 2022) and image matching (Neubert and Schubert 2021).

Based on HDC, HOMS-TC simplifies spectral library matching to efficient cosine similarity searching of HVs. Our HV encoding captures spectral similarity by incorporating peak position and intensity and is tolerant to changes in peak intensity due to instrument errors or noise. Furthermore, the encoding natively supports adding additional reference spectra when the spectral library is updated because each data point is independent. To this end, the proposed algorithm is parallelizable and can be easily accelerated with hardware as they are highly regularized. Thanks to a simplified pipeline and scoring metric, HOMS-TC can be implemented using state-of-the-art hardware accelerators, such as the emerging tensor core units (TCUs). TCUs outperform CUDA cores for matrix multiplication operations and are readily available and programmable in modern NVIDIA GPUs. We evaluate the proposed algorithm using CUDA v11.8 on an NVIDIA GeForce RTX 4090 GPU. Compared with ANN-SoLo, which is a traditional GPU-based OMS solution, HOMS-TC is 31× faster while offering comparable quality as other competing search tools.

## 2 HDC preliminaries

HDC is an emerging computing paradigm that mathematically models features of the neuronal circuits in the human brain by representing data with HVs. Using the following operations, HDC mimics the way human memory works; memorizing information, associating different pieces of information, and understanding relationships between data.

**Reasoning:** The reasoning is done by measuring the similarity between two HVs. For example, we can distinguish between two HVs, $H_{1}$ and $H_{2}$, by examining the similarity between them, i.e. $\delta (H_{1},H_{2})$. If this value is close to zero, indicating that the HVs are nearly orthogonal, then we can conclude that they are distinct. Here, hamming distance and cosine similarity can be used for binary HVs and non-binary HVs, respectively.

**Bundling:** The bundling operation mimics memorization. It is done by element-wise vector addition between HVs. For example, let $H=A_{1}+A_{2}+A_{3}$. H memorizes patterns of HV $A_{1}$, $A_{2}$, and $A_{3}$; $\delta (H,A_{i})\gg 0$ for i∈{1,2,3}. Besides, for randomly generated HV X, δ(H,X)≃0 as H does not contain the information of X.

**Binding:** Using the coordinate-wise vector multiplication (⊗), we can associate different information. This HDC operation is called binding. The resulting HV represents new information, which is nearly orthogonal (dissimilar) to both operand HVs, i.e. δ(A⊗B,A)≃0.

**Flip:** To achieve the desired level of similarity between HVs, we use the flip operation, which involves changing the sign bit of HV elements. For example, flipping D/2 elements of the HV represented by $H={\left\{+1,-1\right\}}^{D}$ produces an HV that is 50% similar to the original HV.

**Permutation:** The permutation operation, $\rho^{n}(H)$ shuffles the components of H with an *n*-bit rotation. The result is a near-orthogonal HV, i.e. $\delta (\rho^{n}(H),H)≃0$ for non-zero *n*.

We can aggregate peak information to HVs with these operations. In the following, we show how HOMS-TC utilizes HDC operations to combine peak data from MS/MS spectra to represent them in an HV format.

## 3 Methods

HOMS-TC is an HDC-based OMS tool that can be parallelized and accelerated with GPU. It consists of two stages: (i) an encoding stage during which MS/MS spectra are converted to HVs and (ii) a search stage that matches query HVs to library HVs to annotate the query spectra.

### 3.1 Data preprocessing and spectra vectorization

**Figure 1.**
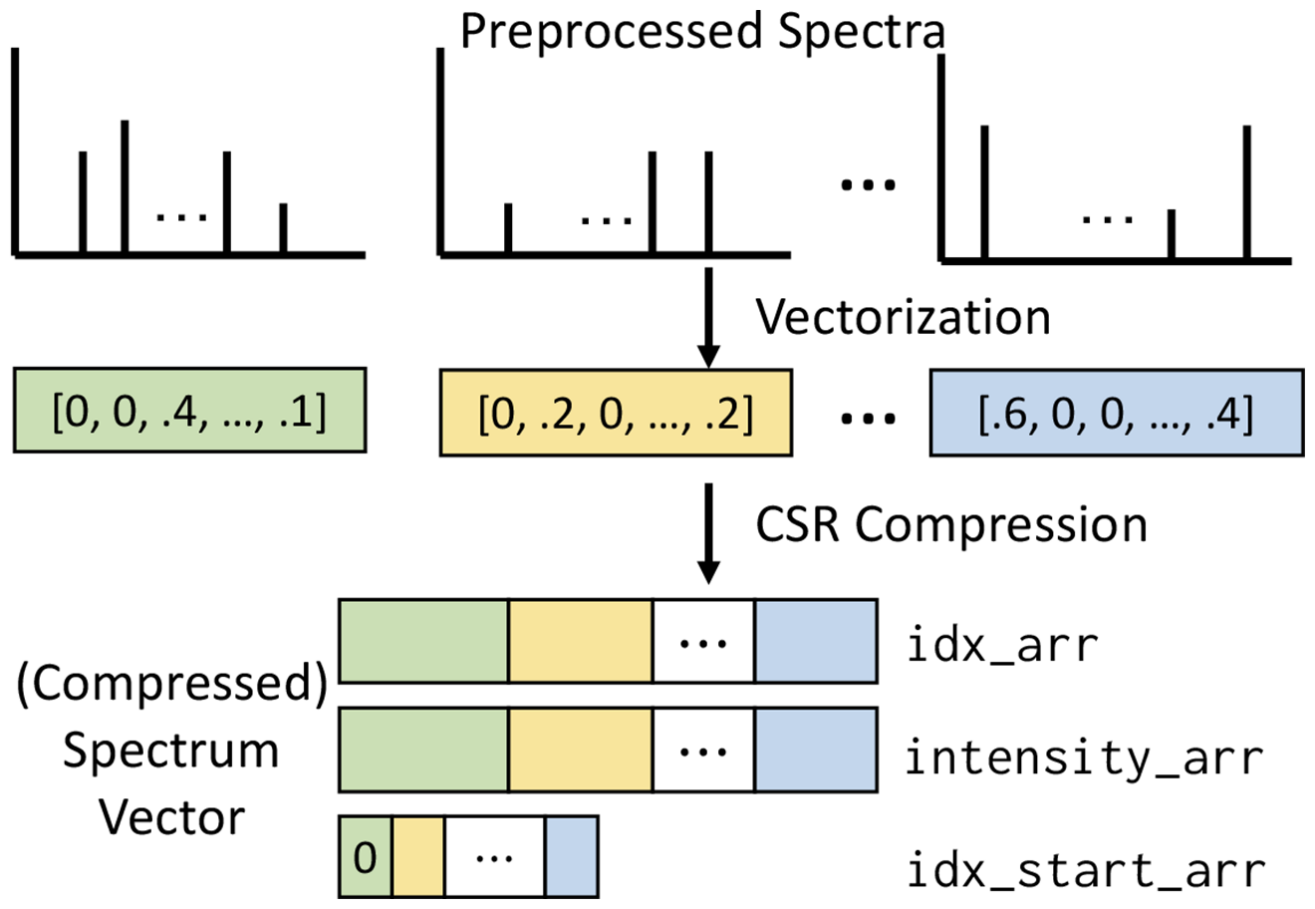
Spectrum vector representation. Preprocessed spectra are converted to spectrum vectors by binning the *m*/*z* range, after which the resulting vectors are compressed using the CSR format.

Before proceeding to the encoding stage, HOMS-TC enhances the search quality by refining spectra using the spectrum_utils package (Bittremieux 2020). The refined spectra are then vectorized and saved in a compressed format (Fig. 1). During refinement, HOMS-TC first limits the mass-to-charge ratio (*m*/*z*) range and removes peaks outside the given *m*/*z* range. Next, low-quality peaks are discarded, including those whose intensity is less than 1% of the most intense peak in the spectrum. Only the *N* most intense peaks are retained, with previous studies (Lam *et al.* 2007, Bittremieux *et al.* 2019) showing that empirically setting *N* between 50 and 150 effectively eliminates noise. Finally, peak intensities are normalized to further enhance the accuracy of the results.

To vectorize spectra, the *m*/*z* range is discretized, resulting in *spectrum vectors—*sparse vectors of floating-point values (intensities). This is realized by dividing the *m*/*z* range into bins and assigning each peak in a spectrum to a bin based on its *m*/*z* value. If multiple peaks fall into the same bin, their intensities are summed up. The bin width determines the level of detail in the vectorized spectrum. A larger bin width leads to a loss of information, while a smaller bin width results in a higher dimensionality for the spectrum vector. For instance, if the mass range is 0−2000 m/*z* and the bin width is 0.04 Da (based on the mass spectrometer resolution), the spectrum vector would have a dimensionality of 50 000. Note that the current HOMS-TC only supports the Da-level bin width, which is ideal for low-resolution ion traps where *m*/*z* errors do not increase with the *m*/*z*.

Vectorizing spectra produce HD, sparse vectors with a sparsity of around 0.1%, which need to be efficiently represented and stored. To accomplish this, we use the compressed sparse row (CSR) format to compress a matrix of spectrum vectors. *The compressed spectrum vector* consists of three one-dimensional arrays: (i) intensity_arr contains peak intensities for all spectra, (ii) idx_arr contains the *m*/*z* bin indexes matching intensity values for all spectra, and (iii) idx_start_arr contains the start indices of each spectrum in the intensity_arr and idx_arr arrays. By doing so, only non-zero intensity values are stored, resulting in optimal data reduction, and individual spectra can be accessed efficiently. During preprocessing, library spectra and query spectra are encoded in a similar manner and saved to disk for efficient reuse in subsequent analyses. Note that HOMS-TC splits spectra based on their precursor charge since matched spectra must have the same charge.

### 3.2 HV encoding

**Figure 2.**
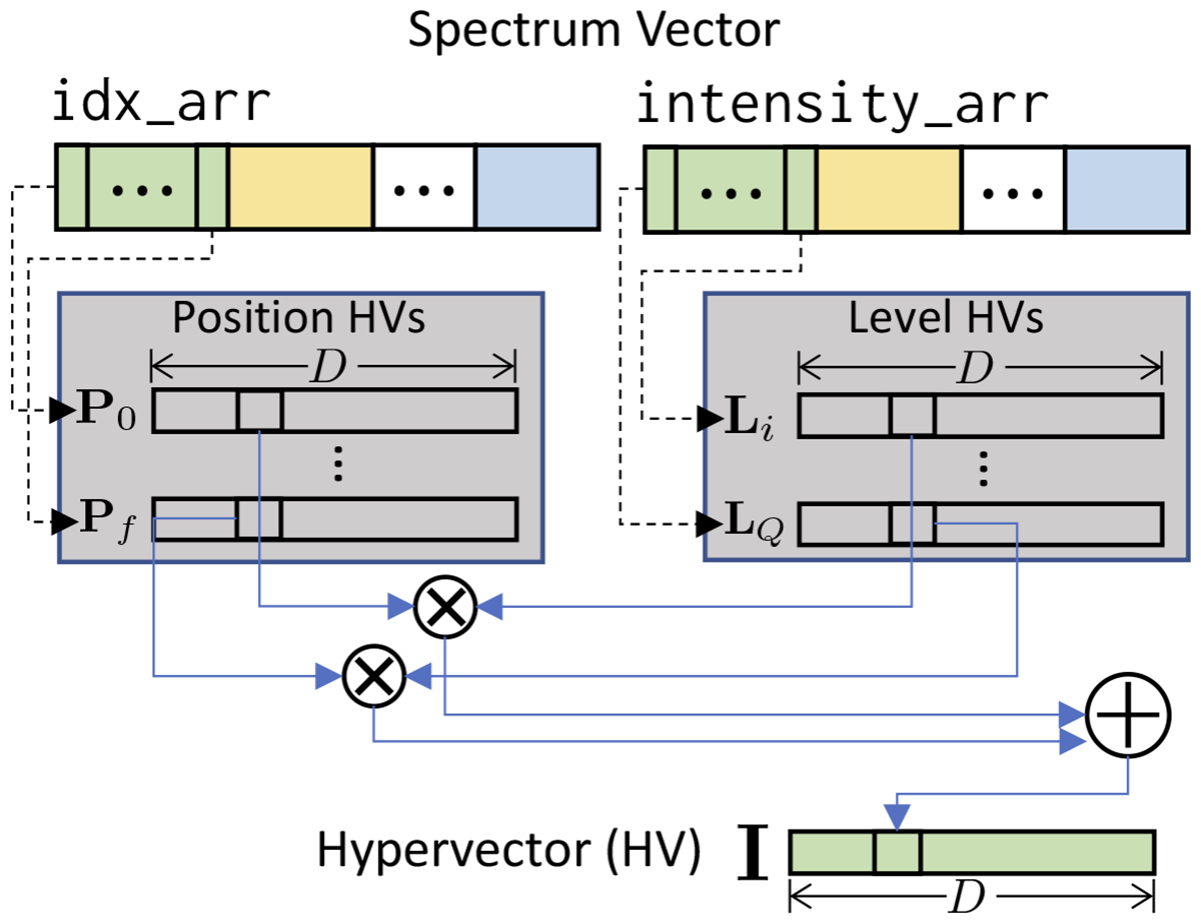
Encoding spectrum vectors to HVs with *D* dimensions using HDC operations. The peak *m*/*z* values are captured by the position HVs $P_{i}$. The peak intensity values are quantized and captured by the level HVs $L_{i}$. The spectrum HVs $I_{i}$ are computed using element-wise multiplication between P and L. The encoding stage is accelerated using CUDA cores.

In the encoding stage, spectrum vectors are converted to HVs. It captures the *m*/*z* values and intensities of peaks and spreads this information over all vector dimensions while also reducing the dimensionality of the original spectrum vectors. The encoding process is illustrated in Fig. 2. First, the *m*/*z* locations of the fragments (idx_arr) are captured using “position HVs” denoted as P. Each bin index is assigned a unique HV $P_{i}$, where $P_{i}$ corresponds to bin *i* and $P_{i}$ is a member of the set $\left\{P_{1},P_{2},\ldots ,P_{f}\right\}$, with *f* being the dimensionality of the spectrum vector. Furthermore, we require that all HVs are orthogonal to each other. Orthogonality helps to ensure that each bin index is represented by a unique position HV, which is important for accurately capturing the *m*/*z* locations of fragments in the spectrum. Without orthogonality, it would be difficult to distinguish between different bin indices, which could lead to errors in the search process. To ensure that $P_{i}$ and $P_{j}$ are orthogonal for i≠j, previous works used a permutation-based method (Salamat *et al.* 2019, 2020). However, $P_{i}$ and $P_{j}$ can be the same when using a permutation-based method if the dimensionality of the HV (*D*) is smaller than that of the spectrum vectors (*f*), for some *i* and *j*. Instead, each element of the position HV P is randomly generated by drawing it from a normal distribution with mean zero and standard deviation one in HOMS-TC. In this way, $P_{i}$ and $P_{j}$ are nearly orthogonal for bin index *i* and *j* where i≠j. In other words, we can distinguish the position of each peak even when f>D.

To capture the peak intensities (intensity_arr), we use “level HVs.” This involves first uniformly discretizing the peak intensities into a finite set of values. Next, we assign a HV $L_{i}$ to each quantization level *i*, with the number of levels *Q*, where i∈[1,Q] and $L_{i}\in \{L_{1},L_{2},\ldots ,L_{Q}\}$. Note that the encoder does not use $L_{0}$ since the intensity of a peak is always greater than 0. Here, the assigned HVs L for each level should reflect the similarity of the corresponding intensities, given that intensity is originally continuous. In other words, the similarity between $L_{i}$ and $L_{i+1}$ should be greater than that between $L_{i+1}$ and $L_{i+x}$ where x>1. The flip operation is used to achieve this closeness. To obtain the level HV $L_{q}$ for a target level *p* among a total of *Q* levels, we flip (D/2)×(q/Q) elements of $L_{1}$. Note that reflecting intensity similarities using level HVs in this way is robust to changes in peak intensities, e.g. due to experimental variability.

Finally, given a set of position HVs P and level HVs L, we aggregate peak information in a spectrum into an HV by binding the HVs P and L, which correspond to peak positions and intensities, respectively. In turn, we bundle those results to memorize patterns in a spectrum HV I as follows: with P the set of tuples (i,j), where *i* is the peak index and *j* the quantized level of its intensity in the spectrum vector. In this fashion, both the positional information and intensity of each peak are captured and aggregated into the spectrum HV I. A key aspect of the HV representation is its robustness to spectrum variability. For varying peak intensities, only a few elements (fewer than the HV’s dimensionality) in the level HV will change. Hence, the similarity between the HVs of matched reference and query pairs remains stable. Additionally, if peaks are added or removed, the bundling operation used to memorize the peak information in I ensures that the remaining peak information is memorized, and thus the similarity is well-preserved.


(1) $$I=\sum_{(i,j)\in P}P_{i}⊗L_{j},$$

Unlike the existing HDC encoding method, ID-Level encoding (Imani *et al.* 2017), the proposed encoder skips the spectrum vector component with zero values. The capture of these values works as an offset in the HV. Exploiting the sparsity of the spectrum vector reduces the number of additions dramatically. Since there are up to 150 peaks per spectrum (after preprocessing) and 30 000 bins in most cases, the required number of addition is reduced by one or two orders of magnitude.

### 3.3 Spectral library search

HOMS-TC uses a two-step cascade search strategy (Kertesz-Farkas *et al.* 2015) to optimize the identification of unmodified and modified peptides while controlling the false discovery rate (FDR). During the first step, unmodified peptides are identified using a small precursor *m*/*z* tolerance, while during the second step, a wide precursor *m*/*z* tolerance is used to identify modified peptides using OMS. FDRs are computed after each step based on the target-decoy strategy (Elias and Gygi 2007). During the first step of the cascade search, FDR calculations proceed in the standard fashion. In contrast, during the second step of the cascade search, a subgroup FDR strategy (Fu 2012, Fu and Qian 2014) is used to capture variations in the score distributions incurred by different PTMs (Bittremieux *et al.* 2018).

**Figure 3.**
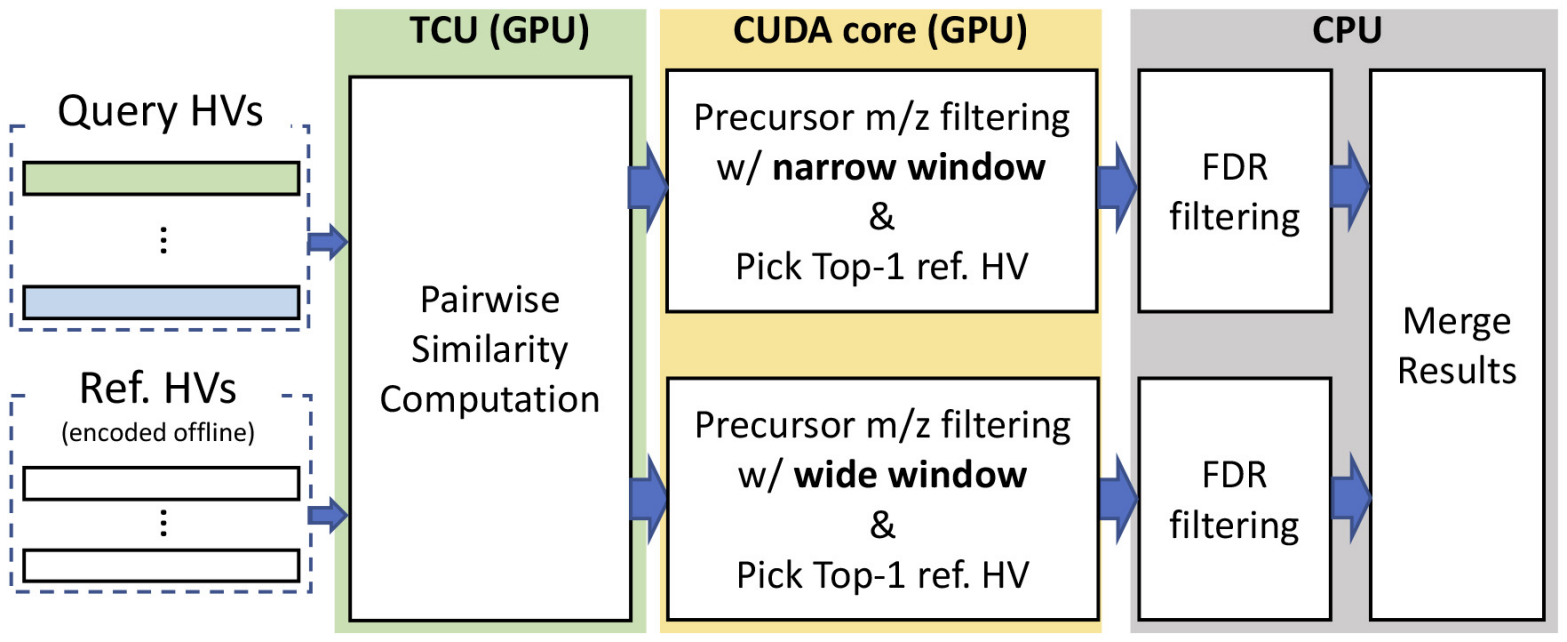
Search stage in HOMS-TC. TCUs and CUDA cores perform similarity computation and search, respectively. Search using two window conditions is done in parallel.

Figure 3 illustrates the flow of the spectrum search, including selecting candidates from the spectral library based on the precursor *m*/*z* tolerance and the pairwise similarity computations. Both library and query spectra are encoded into HVs, which are used to identify the best match between a query spectrum and a library spectrum based on cosine similarity. Note that we use the same similarity value for two levels. As such, for efficiency, HOMS-TC processes *both levels of the cascade search in parallel* and the accepted identifications are merged at the end. In other words, HOMS-TC overlaps the two levels of cascade search, thereby avoiding redundant computations and maximizing parallelism.

### 3.4 Acceleration using GPUs

Two stages of HOMS-TC are parallelizable using GPUs. The encoding stage can be parallelized across HV dimensions and datapoints as they are independent of each other. It can be implemented in a similar way to existing GPU-based HDC framework (Kang *et al.* 2022a,b). The CUDA cores in the GPU process the encoding and store spectrum HVs in the GPU global memory. For the encoding stage, HOMS-TC generates HVs for query and reference spectra on-the-fly, not saving the encoded results to the disk. Like other tools, e.g. ANN-SoLo (Bittremieux *et al.* 2018, 2019), the encoded reference HVs can be reused. However, our results show that generating HVs in place is faster due to the data transfer time from the disk to the host memory and from the host memory to GPU memory.

**Figure 4.**
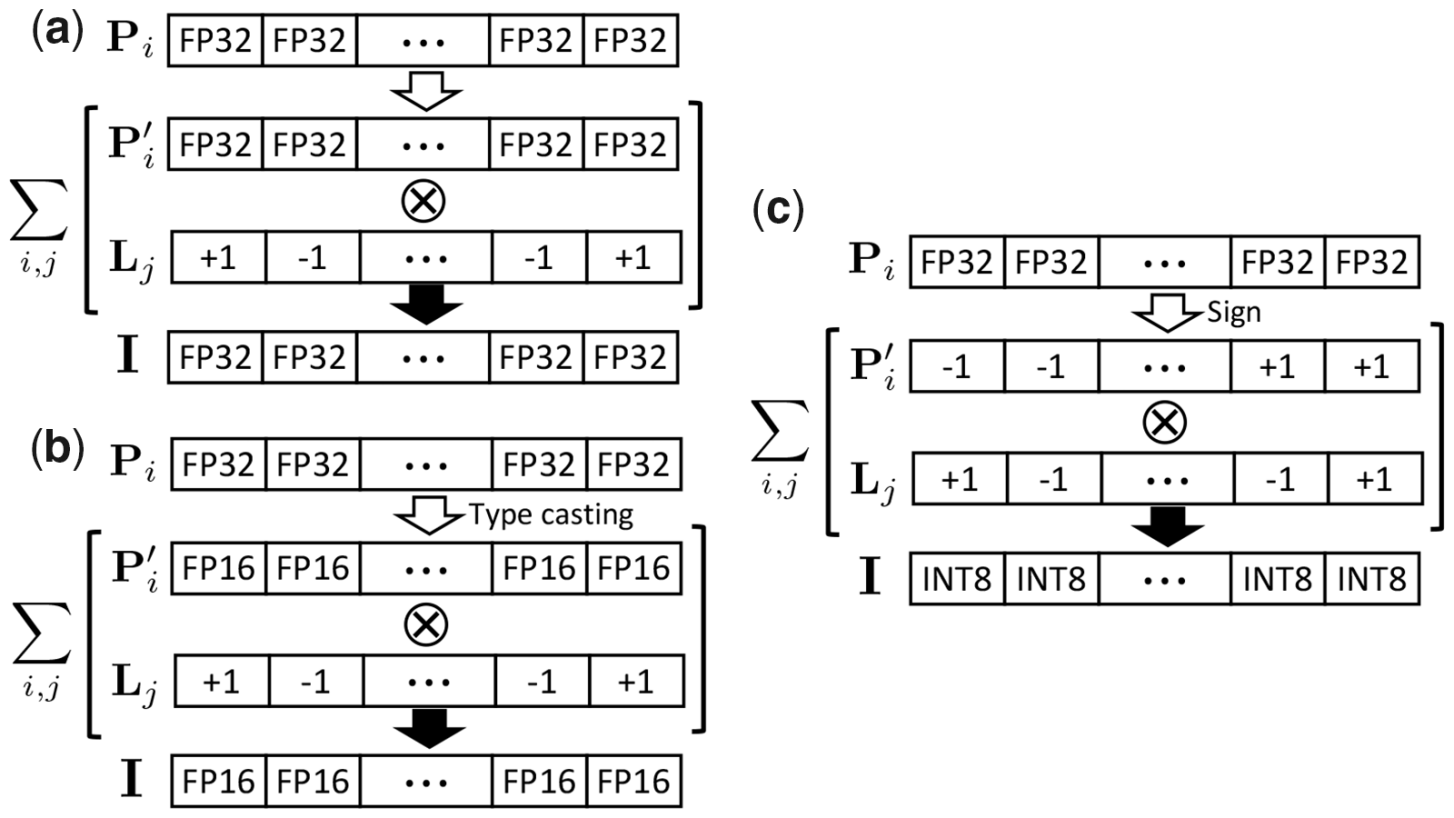
Various spectrum HV precision used in HOMS-TC. (a) FP32 precision, (b) FP16 precision, and (c) INT8 precision. Depending on the precision, HOMS-TC is run on either CUDA cores or TCUs.

The search stage consists of score calculation and top-1 reference HV search (Fig. 3). Here, the score calculation is inherently equivalent to matrix multiplication. Recent GPUs have TCUs that are specialized for such operations, which can be easily used with CUTLASS (Kerr *et al.* 2022) or the CUBLAS (NVIDIA 2022) application programming interface. By using TCUs to calculate similarity scores, throughput can be maximized. Note that using TCUs does not result in data movements since TCUs and CUDA cores share the same GPU global memory. However, to exploit TCU hardware, several constraints need to be satisfied. First, the HV dimensionality and the batch size need to be multiples of 8. Also, the choice of HV precision is limited. Using half-precision floating-point (FP16) or eight-bit integer (INT8) precision is required. In HOMS-TC, the precision determines the amount of information that the HVs can represent, which can affect search quality. Figure 4 illustrates how HOMS-TC can manage the precision of the encoded HVs. FP16 spectrum HVs can be obtained by using FP16 position HVs. To use INT8 precision for I, we binarize the position HV by taking the sign bit for each component. The level HV is ${\left\{-1,1\right\}}^{D}$. If there are up to 50 peaks per spectra, each component of the spectrum HV is in the range between −50 and +50, satisfying the range of INT8 ([−128, 127]). Empirically, we found that INT8 is enough to get sufficient search quality. We discuss the impact of precision in Section 4.3.1.

The top-1 reference HV search part is accelerated using CUDA cores. We use a conventional parallel maximum reduction strategy (Harris 2007) while satisfying precursor *m*/*z* tolerance conditions. HOMS-TC targets OMS with cascade search; searching with two precursor *m*/*z* conditions runs in parallel. Finally, the CPU processes subsequent FDR filtering and merges the results from both levels of the cascade search.

## 4 Results

### 4.1 Experimental setup

#### 4.1.1 System configuration

The evaluation was conducted on a system with an Intel i7-11700K CPU and 64 GB of RAM, and NVIDIA Geforce RTX 4090 with 24 GB of VRAM and fourth-generation TCUs. The HOMS-TC algorithm was implemented on the GPU using a state-of-the-art GPU-based HDC framework (Kang *et al.* 2022a,b). Due to the limited memory on the GPU, the reference and query data were divided into batches. The batch size was set to use the maximum amount of VRAM available for GPU-based solutions.

#### 4.1.2 Dataset

We tested the performance of HOMS-TC on two real-world datasets. The first dataset consists of the iPRG2012 dataset (Chalkley *et al.* 2014) with 17 993 query spectra, which was searched against a combined reference library derived from a TripleTOF yeast spectral library (Selevsek *et al.* 2015) and a human HCD spectral library compiled by NIST (version 2016/09/12), totaling 1 188 168 library spectra. To create the spectral library, we removed decoy hits from the yeast spectral library and concatenated it to the human HCD spectral library using SpectraST (Lam *et al.* 2007). Additionally, duplicates were removed and decoy spectra were added in a 1:1 ratio using the shuffle-and-reposition method (Lam *et al.* 2010).

The second dataset consists of HEK293 data from 24 peak files, with on average 46 715 spectra per file (Chick *et al.* 2015). These data were searched against the MassIVE-KB spectral library (version 2017/11/27) (Wang *et al.* 2018), which was compiled using repository-scale reprocessing of public human proteomics data on MassIVE, and contains 3 009 902 spectra. Library spectra were processed as described above to remove duplicate spectra and add decoy spectra.

The query and library spectra were preprocessed using the hyperparameters listed in Table 1. Additionally, low-quality spectra with less than 10 peaks and a 250 *m*/*z* mass range were discarded, and peaks within a 0.05 *m*/*z* window around the precursor *m*/*z* were removed. After preprocessing, there were 15 867 query spectra and 1 162 392 library spectra in the iPRG2012 dataset remaining. For the HEK293 dataset, on average 46 665 query spectra per peak file remained, as well as 2 992 672 library spectra. All MS data, spectral libraries, preprocessed spectra, and identification results are available on the MassIVE repository with the dataset identifier MSV000091183.

**Table 1.** Parameters used for the evaluation.

	Dataset	
Parameter	iPRG2012	HEK293
Max peaks per spectrum	50	
Min/Max *m*/*z*	101/1500	
Bin size	0.05 Da	0.04 Da
Precursor *m*/*z* tolerance (narrow)	20ppm	5ppm
Precursor *m*/*z* tolerance (wide)	500 Da	500 Da

### 4.2 Baselines

We evaluate HOMS-TC in terms of search quality and speed improvement. Also, we show HOMS-TC performance according to the three key parameters in HDC: HV precision, HV dimensionality, and quantization level. We compare them to those obtained using existing search tools, including the state-of-the-art OMS solution running on GPU, ANN-SoLo GPU v0.3.3 (Bittremieux *et al.* 2019), and the CPU-based OMS tools, ANN-SoLo CPU v0.3.3 (Bittremieux *et al.* 2018), MSFragger v3.3 (Kong *et al.* 2017), and SpectraST v5.0 (Lam *et al.* 2007, Ma and Lam 2014). For ANN-SoLo, we set the number of clusters (*C*), the number of clusters to visit (*N*), and batch size (*B*) to 512, 128, and 8192, respectively, to fully utilize GPU memory in the HEK293 dataset case. The same *C*, *N*, and *B* are used for all datasets for fair speed comparison. We evaluated all search results at a fixed FDR threshold of 1% using Pyteomics (Goloborodko *et al.* 2013). Note that we compare the speed of HOMS-TC to ANN-SoLo only, which is currently the fastest OMS tool. We sum all the execution times for each precursor charge. For the HEK293 dataset, we run the search tool for all query files and compute an average of the runtime. Also, we assume that the preprocessing was done offline, as this only needs to be done once.

### 4.3 Impact of HV configuration

#### 4.3.1 HV precision

The precision of the HV impacts the amount of information they are able to represent. While limited precision has the advantage that the data can be compressed more efficiently and specific precision such as FP16 and INT8 can enable the use of TCUs, it may reduce the search quality. We evaluate the search quality and the runtime according to the precision of the spectrum HVs, I. Here, we fixed the HV dimension and quantization level to 8192 and 32, respectively. As shown in Supplementary Table S1, using FP16 or INT8 offers similar search quality to the single-precision floating-point (FP32) case. However, the spectral library search stage in HOMS-TC can be accelerated with TCUs when using FP16 and INT8 spectrum HV. Thanks to the TCU acceleration, using INT8 (FP16) can accelerate the search stage by up to 3.5× (2.4×) and the end-to-end HOMS-TC by up to 2.9× (2.1×) compared with the FP32 case that uses CUDA cores (Supplementary Fig. S1). Since at most 50 peaks exist in our evaluation, we use INT8 precision in HOMS-TC, considering the search quality and performance tradeoff.

#### 4.3.2 HV dimensionality

Another important hyperparameter that allows a trade-off between search results and runtime is the HV dimensionality (*D*). Because the time complexity of the cosine similarity calculations is proportional to *D*, a higher dimensionality will lead to increased runtimes (Supplementary Fig. S2). In contrast, an overly low dimensionality limits the fidelity with which the HVs can encode the spectral data, negatively impacting the identification performance. We can clearly observe the influence of *D* on the identification performance (Supplementary Table S2). Note that we use INT8 HV and 32 quantization level. Optimal identification performance is achieved at high *D*, while the number of identified spectra decreases for lower HV dimensionalities. Therefore, a sufficiently high HV dimensionality is required to achieve a good search quality. We use D=8192 since search quality is saturated at this value.

#### 4.3.3 Quantization level

High quantization levels may not be flexible to the peak intensity changes due to noise and PTMs. A low quantization level leads to low resolution in intensity capturing of the encoder. To observe the impact of *Q*, we fixed *D* to 8192 and HV precision to INT8. We set the quantization level *Q* from 8 to 64 and measured the search quality. As shown in Supplementary Table S3, the search quality is less sensitive to *Q*. We set the default value for the *Q* to 32.

### 4.4 Search quality

**Figure 5.**
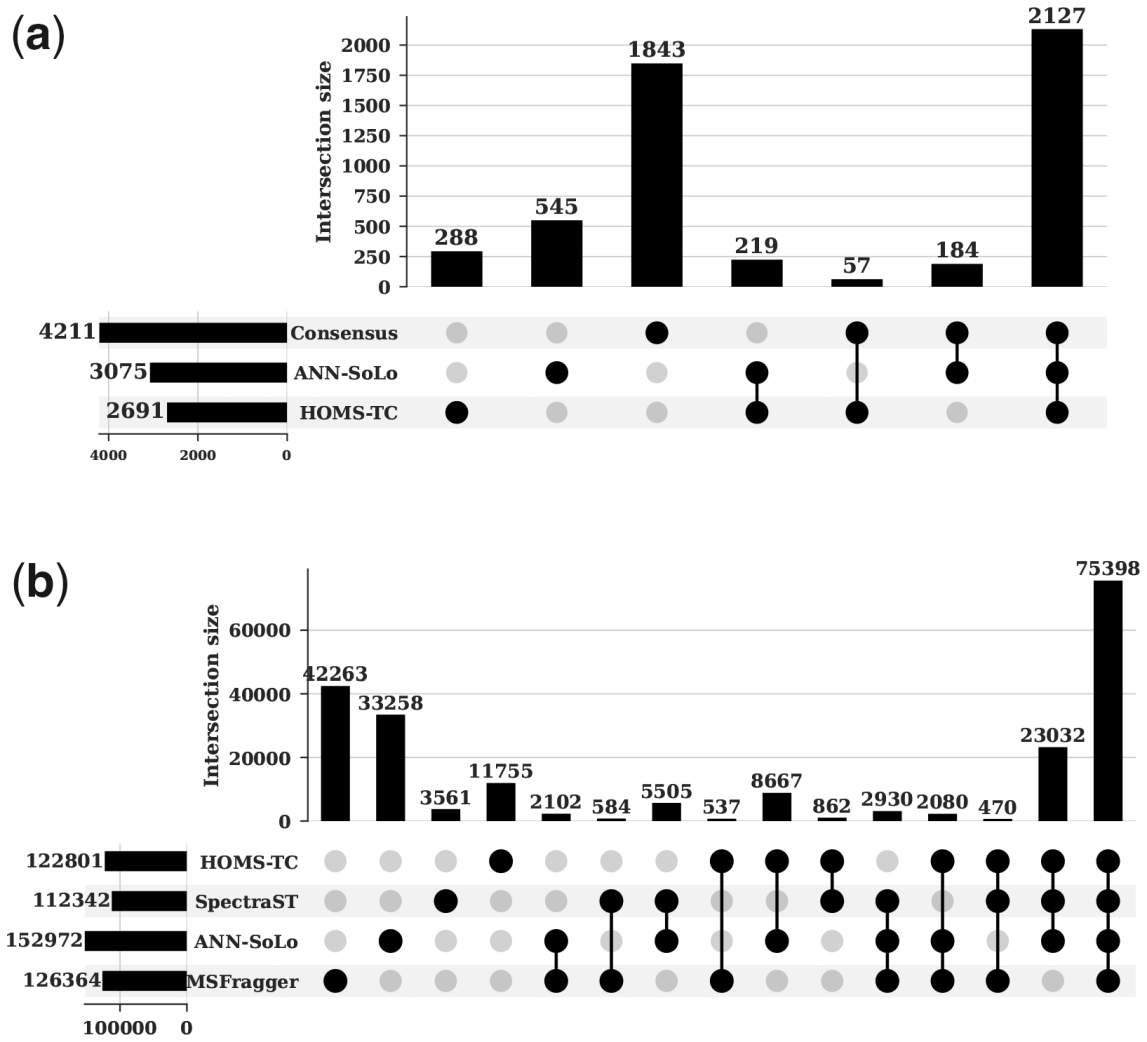
Comparison of the search quality (the number of unique peptides) of HOMS-TC to baselines visualized with UpSet plot. (a) HOMS-TC versus ANN-SoLo and consensus results on iPRG2012 dataset. (b) HOMS-TC versus baseline identification results on HEK293 dataset.

To compare search quality, we count the number of identified spectra after FDR filtering. Note that the high-performing (in general) hyperparameters are used for HOMS-TC: HV precision, HV dimensionality (*D*), and quantization level (*Q*) are set to INT8, 8192, and 32, respectively. For the iPRG2012 dataset, we compare the search results to the consensus identifications from multiple participants of the iPRG2012 study (Chalkley *et al.* 2014). The analysis result shows that HOMS-TC is able to correctly identify around 60% of identical spectra of the iPRG2012 consensus results (see Fig. 5a). Although HOMS-TC identifies slightly fewer spectra than ANN-SoLo, the extra ANN-SoLo identifications predominantly do not match or conflict with the iPRG2012 consensus results, and thus are presumably less reliable.

Additionally, we compare the search results of HOMS-TC to ANN-SoLo, MSFragger, and SpectraST using the larger HEK293 dataset. As no ground truth is available for the HEK293 dataset, we compare the number of identified spectra and unique identified peptides from HOMS-TC and baseline OMS tools, SpectraST (Lam *et al.* 2007, Ma and Lam 2014), ANN-SoLo (Bittremieux *et al.* 2018, 2019), and MSFragger (Kong *et al.* 2017). The results indicate that HOMS-TC identified a similar number of spectra as SpectraST and MSFragger. Although ANN-SoLo managed to identify more spectra, a significant number of identifications were only unique to ANN-SoLo, while the number of spectra commonly identified by HOMS-TC, ANN-SoLo, and SpectraST was similar (see Fig. 5b). Meanwhile, a high portion of identifications from MSFragger is unique or conflicting matches compared with ANN-SoLo, SpectraST, and HOMS-TC. MSFragger adopts a different approach to perform OMS, a sequence database search, and it uses a sequence database containing the entire human proteome. These results clearly demonstrate that HOMS-TC achieves a similar spectrum identification performance as alternative spectral library search engines.

**Figure 6.**
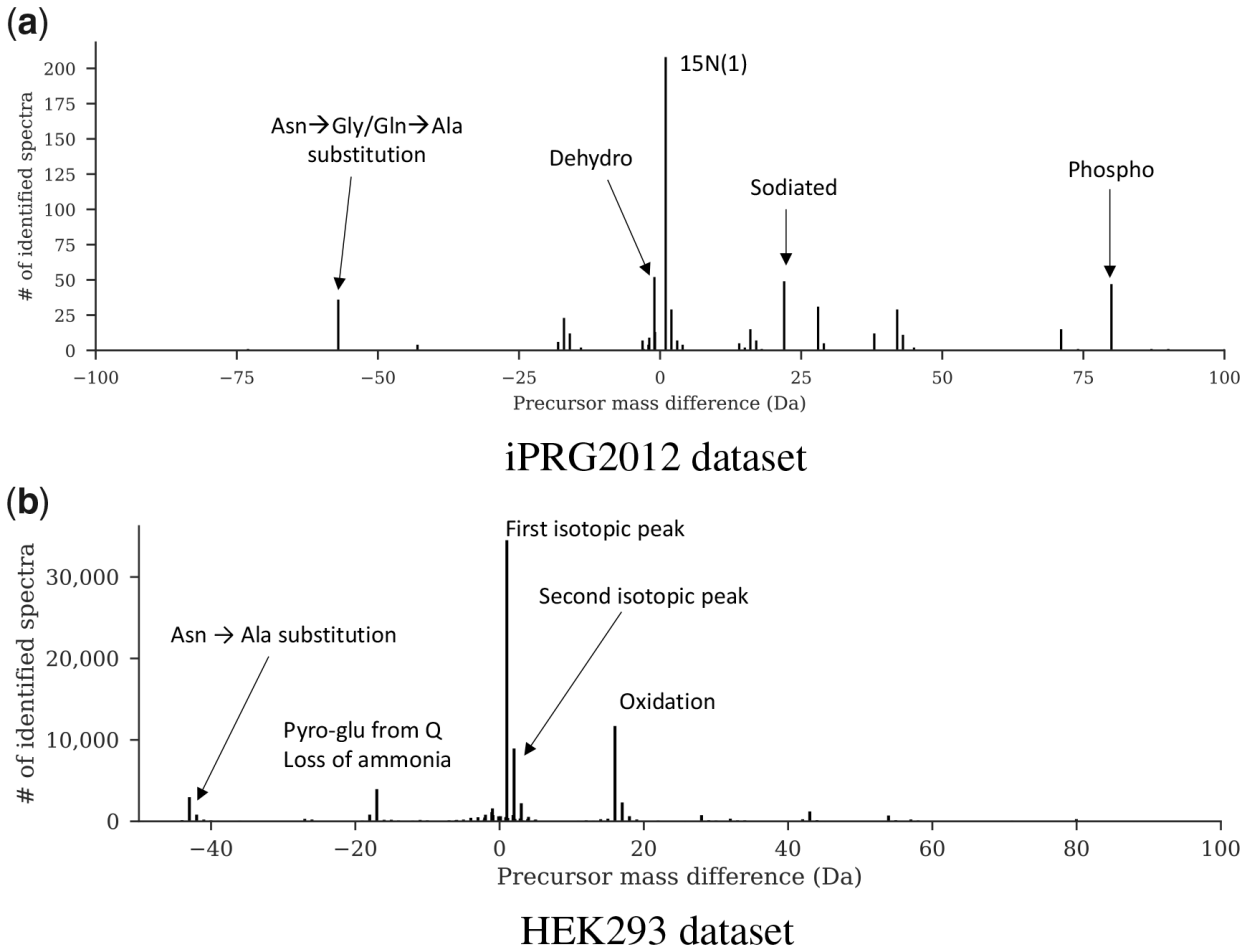
Precursor mass difference of unique identified peptides from HOMS-TC. Only non-zero precursor mass differences are visualized and the five most frequent modifications are annotated. (a) iPRG2012 dataset. (b) HEK293 dataset.

Figure 6 shows the possible modifications on the protein sample based on the precursor mass difference of the identified spectra. Specifically, we compute the precursor mass difference, collect only modified peptides, and compare results with the Unimod public database of protein modifications (Creasy and Cottrell 2004). For example, for the HEK293 dataset, the mass shift caused by various amino acid substitutions, such as Asn to Ala and Phe to Tyr, was observed. Also, HOMS-TC detected chemical changes such as oxidation, and hydroxylation, as well. The modification, including GlyGly, acetylation, and phosphorylation, was also observed at a lower rate. This implies that HOMS-TC can identify biologically relevant PTMs through the OMS. Note that HOMS-TC does not determine modification identities, but they can be obtained from the precursor mass difference using post-processing tools like PTM-Shepherd (Geiszler *et al.* 2021) and AA_stat (Levitsky *et al.* 2021).

### 4.5 Speed improvement and analysis

We compare the runtime of HOMS-TC to ANN-SoLo using GPU acceleration (Bittremieux *et al.* 2019), which offers the fastest spectral search speed among competing tools. ANN-SoLo first needs to build an index or encode the original data, which can be reused for subsequent processing. For the first run of ANN-SoLo, which includes the reference indexing time, HOMS-TC is 32× faster than ANN-SoLo GPU on average. When indexes are reused on ANN-SoLo, HOMS-TC achieves 35× and 28× speedup on iPRG2012 and HEK293 dataset, respectively, resulting in 31× speedup on average (geomean) over the baseline (see Table 2). Due to massive parallelization and simplified pipeline, the search stage itself is 51× faster on average. Note that we averaged the execution time of run for each query file on the HEK293 experiment. In other words, HOMS-TC generates the reference HVs for every run in an on-the-fly manner. The baseline tool does not find the most similar spectra directly. Instead, it first reduces the search space and subsequently computes similarity scores, with this final score calculation step amounting to 53% of the total search time on average. In contrast, HOMS-TC performs a one-pass search, leading to a significant speedup. Also, while ANN-SoLo uses CUDA cores with FP16 precision during the candidate selection, the proposed tool utilizes TCUs with lower precision (INT8).

**Table 2.** HOMS-TC speedup over the state-of-the-art high-performance OMS tool [ANN-SoLo GPU (Bittremieux *et al.* 2019)].

	End-to-end (second run)		Reference encoding (indexing)	
	iPRG2012	HEK293	iPRG2012	HEK293
ANN-SoLo	72.77s (1×)	290.08s (1×)	12.01s (1×)	20.82s (1×)
HOMS-TC	2.08s (35×)	10.4s (28×)	0.95s (12.6×)	2.21s (9.4×)

In the HEK293 experiment, the runtime was averaged over the execution of each query file.

Meanwhile, as spectral libraries are continuously growing, their efficient indexing of them is significant. HOMS-TC improves the indexing time by parallelizing across HV dimensions and datapoints, which can be accelerated with GPUs. As shown in Table 2, the encoding of reference spectra using HOMS-TC shows 10.9× speedup on average compared with the baseline. Note that the encoding of new datapoints in HOMS-TC is independent of previous data, which can be easily parallelized.

**Figure 7.**
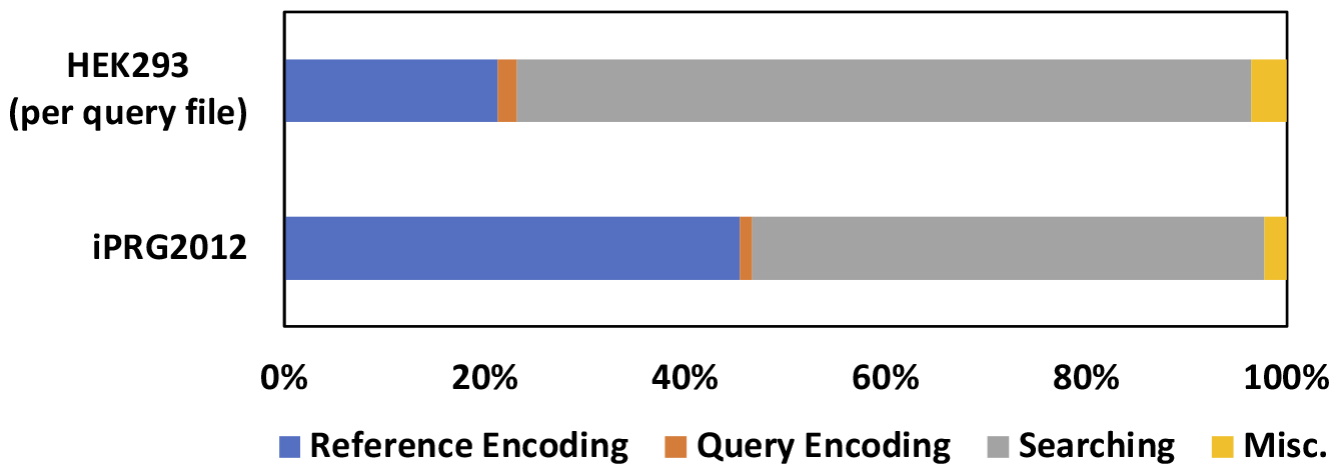
HOMS-TC runtime breakdown by stage. On larger datasets, the portion of the search stage increases.

According to the profiling results, we found that during the encoding stage and the search stage, 70% of the CUDA core and 95.01% of the TCU were utilized, respectively. The entire HOMS-TC process was executed on GPUs, resulting in high hardware utilization when compared with ANN-SoLo GPU (Bittremieux *et al.* 2019), which only utilizes GPUs for the nearest neighbor search step. Figure 7 illustrates the breakdown of HOMS-TC by stage. The miscellaneous section includes the time taken for GPU memory allocation and memory copying from the CPU (host) to the GPU.

The encoding of query spectra has a low impact on the overall runtime, accounting for only 1.5% of the total execution time on average. The search stage’s contribution to the total runtime increases from 51% to 73% in the HEK293 dataset. Encoding reference spectra takes up 33% of the total runtime on average. HOMS-TC encodes the reference spectra on-the-fly, but this can be minimized when reference HVs are reused, like the HEK293 dataset that shares the same reference database for multiple query files.

### 4.6 Discussion and future work

In order to further improve the search quality, an advanced encoding method that takes into account other OMS characteristics can be developed. One potential approach is to use population-based discretization of peak intensities, similar to MyriMatch (Tabb *et al.* 2007), to generate level HVs. Additionally, we can consider various peak changes resulting from PTMs, such as peak shifts during the encoding.

Two approaches can accelerate HOMS-TC further. First, since the search stage is mainly handled by TCUs, we expect that a future GPU with the next-generation TCU can enhance the performance of our tool. For example, when compared with HOMS-TC running on NVIDIA Geforce RTX 3090 with previous (third) generation TCU, we can get 1.8× speedup on the search stage using the latest GPU used in the evaluation. Secondly, we can scale HOMS-TC to multiple GPUs. We have demonstrated the spectral library search acceleration on a single GPU in this article, and it runs in a batched manner due to memory capacity limitation and limited TCUs. Exploiting multiple GPUs with TCUs can offer more parallelism and lead to execution time reduction; this remains our future work.

## 5 Conclusion

In this article, we proposed an HDC-based OMS solution accelerated with TCUs dubbed HOMS-TC. HOMS-TC encodes spectra into HVs that reflect the position and intensity of peaks and are tolerant to peak intensity changes. We enable OMS in one shot. Every dimension of the HVs can be computed independently, which makes the algorithm easily parallelizable. As HOMS-TC employs a simplified OMS pipeline and cosine similarity metric with reduced precision, the algorithm can be accelerated with TCUs. In addition, our proposed encoding method achieves 10.9× speedup on average, which implies that HOMS-TC can effectively handle the constantly growing spectral libraries. The evaluation results show that HOMS-TC offers comparable search quality to existing OMS tools, with 31× faster execution time on average compared with the state-of-the-art tool running on GPU.

## Supplementary Material

btad404_Supplementary_Data

## Data Availability

The data underlying this article will be shared on reasonable request to the corresponding author.
