# Supplementary material for: Accelerating open modification spectral library searching on tensor core in high-dimensional space
Source: Bioinformatics. 2023 Jun 27;39(7):btad404. doi: 10.1093/bioinformatics/btad404 (PMC10323168; doi:10.1093/bioinformatics/btad404)
Supplement: btad404_Supplementary_Data [file btad404_supplementary_data.pdf]

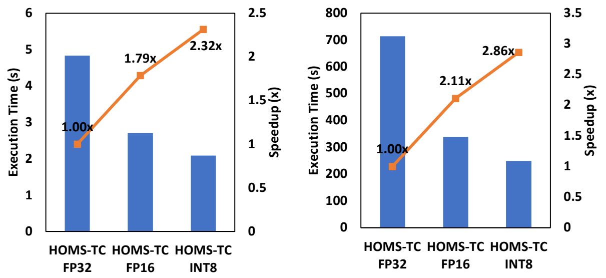

(a) iPRG2012 dataset. (b) HEK293 dataset.

Fig. S1: The execution time of HOMS-TC according to the HV precision.

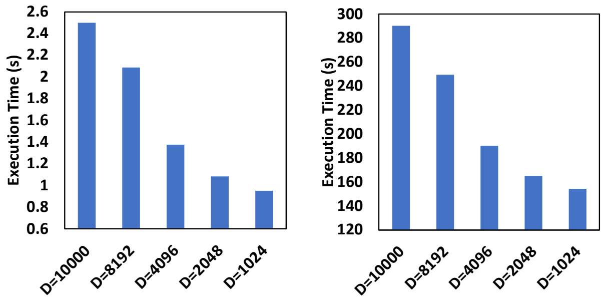

(a) iPRG2012 dataset. (b) HEK293 dataset.

Fig. S2: The execution time of HOMS-TC according to the HV dimensionality.

Table S1. Search quality of HOMS-TC according to HV precision

	# of Identified Spectra (Open Search Matches)		# of Unique Peptides (Open Search Matches)	
	iPRG2012	HEK293	iPRG2012	HEK293
HOMS-TC FP32	4537 (1520)	453090 (130703)	2742 (1195)	256313 (97798)
HOMS-TC FP16	4347 (1330)	456453 (134066)	2661 (1069)	259608 (99810)
HOMS-TC INT8	4440 (1337)	457097 (130085)	2691 (1074)	259070 (97431)

Table S2. Search quality of HOMS-TC according to HV dimensionality D

	# of Identified Spectra (Open Search Matches)		# of Unique Peptides (Open Search Matches)	
	iPRG2012	HEK293	iPRG2012	HEK293
$D = 10000$	4468 (1354)	458375 (131329)	2705 (1058)	260010 (97045)
$D = 8192$	4440 (1337)	457097 (130085)	2691 (1074)	259070 (97431)
$D = 4096$	4284 (1377)	452947 (128960)	2591 (1069)	257003 (96725)
$D = 2048$	4053 (1273)	441822 (122231)	2515 (1021)	252559 (92173)
$D = 1024$	3640 (1094)	421448 (111540)	2294 (909)	243337 (85917)

Table S3. Search quality of HOMS-TC according to quantization level Q

	# of Identified Spectra (Open Search Matches)		# of Unique Peptides (Open Search Matches)	
	iPRG2012	HEK293	iPRG2012	HEK293
$Q = 8$	4452 (1430)	455422 (129320)	2710 (1127)	259003 (96930)
$Q = 16$	4445 (1407)	458658 (131866)	2695 (1108)	259602 (98437)
$Q = 32$	4440 (1337)	457097 (130085)	2691 (1074)	259070 (97431)
$Q = 64$	4418 (1381)	456419 (129367)	2677 (1082)	259283 (96308)

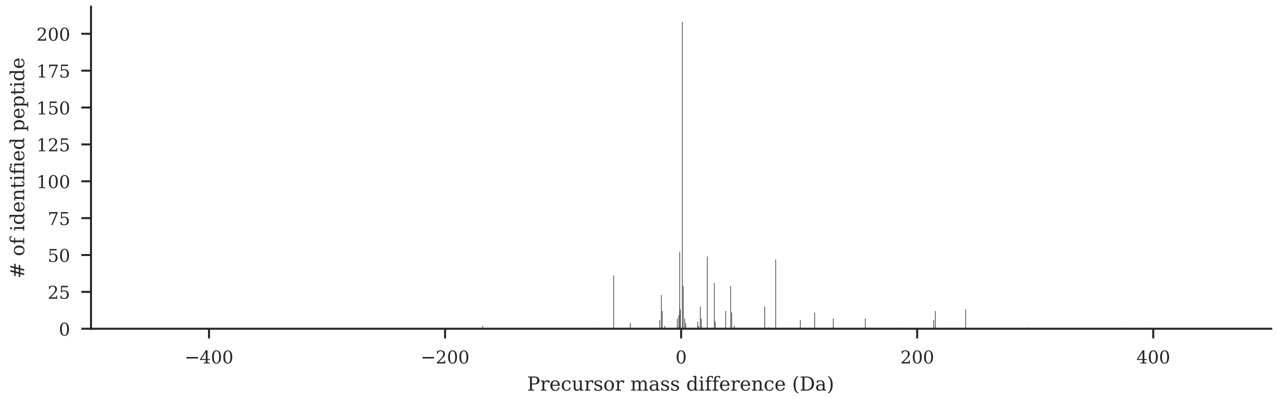

(a) iPRG2012 dataset

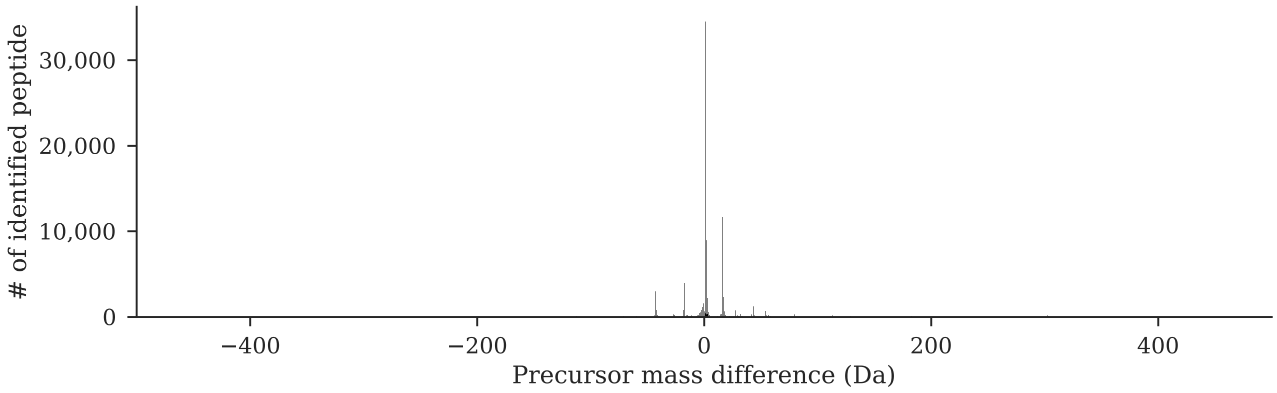

(b) HEK293 dataset

Fig. S3: Precursor mass difference of unique identified peptides from HOMS-TC across the entire mass range window (-500Da – +500Da)

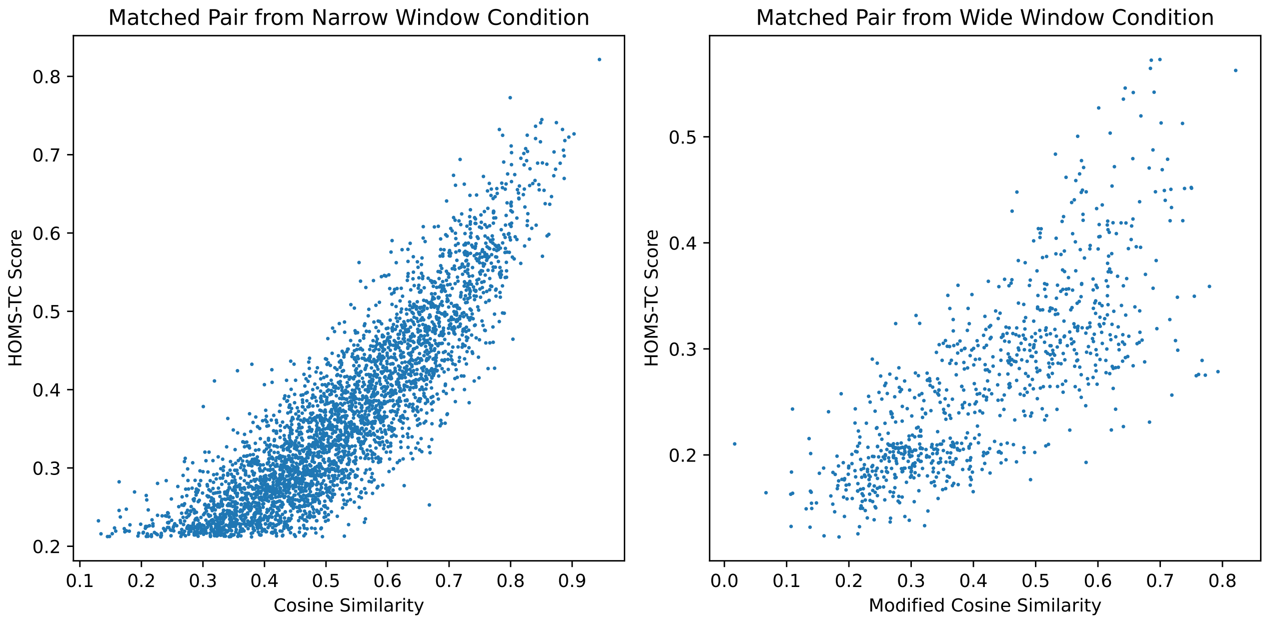

(a)

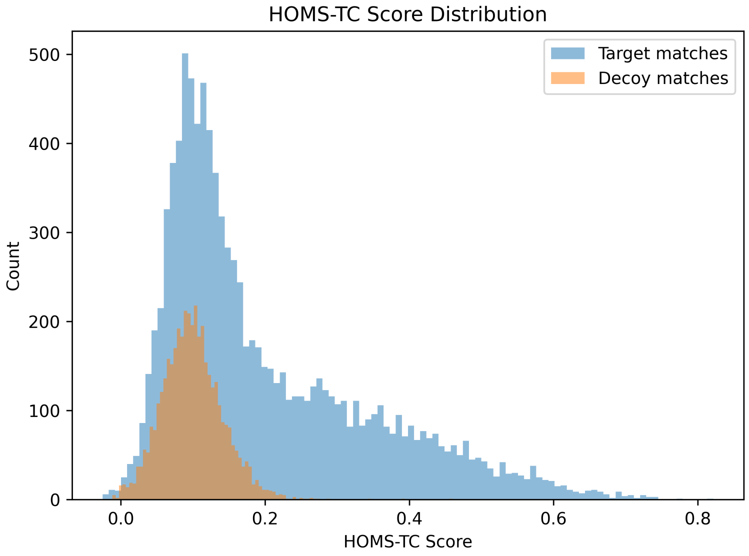

(b)

Fig. S4: Score analysis of HOMS-TC. (a) The left figure displays the correlation between HOMS-TC scores and the cosine similarity scores of the matched pairs from the standard search. The right figure shows the correlation between HOMS-TC scores and the modified cosine similarity scores of the matched pairs from the open modification search. The scatter plots clearly demonstrate that the HOMS-TC score is correlated with conventional score metrics. (b) A density histogram of HOMS-TC scores for matching target and decoy spectra. The figure shows the expected bimodal distribution of the target scores, with the low-ranking scores overlapping with the decoy scores.
